# Guideline adherence in hospital recruited and population based COPD patients

**DOI:** 10.1186/s12890-018-0756-8

**Published:** 2018-12-20

**Authors:** Bahareh Jouleh, Marta Erdal, Tomas Mikal Eagan, Per Bakke, Amund Gulsvik, Rune Nielsen

**Affiliations:** 1 Department of Thoracic Medicine, Haukeland University Hospital, Bergen, Norway; 2 Department of Clinical Science, University of Bergen, N-5021 Bergen, Norway; 3 Haukeland, Universitetssjukehus, Laboratoriebygget, Jonas Lies veg 87, 5021 Bergen, Norway

**Keywords:** Chronic obstructive pulmonary disease, Guidelines, Adherence, General population

## Abstract

**Background:**

Evidence from several studies show poor guideline adherence to COPD treatment, but no such study has been undertaken in Norway. The objectives of this study, was to estimate and compare the guideline adherence to COPD treatment in general population-based and hospital-recruited COPD patients, and find possible predictors of guideline adherence.

**Methods:**

From the prospective, observational EconCOPD-study, we analysed guideline adherence for 90 population-based COPD cases compared to 245 hospital-recruited COPD patients. Overall guideline adherence was defined as correct pharmacological treatment, and influenza vaccination the preceding year, and having received smoking cessation advice. Multivariate logistic regression analysis was performed with the dichotomous outcome overall guideline adherence adjusting for relevant variables.

**Results:**

The overall guideline adherence for population-based COPD cases was 6.7%, significantly lower than the 29.8% overall guideline-adherence amongst hospital-recruited COPD patients. Adherence to pharmacological treatment guidelines was 10.0 and 35.5%, for the two recruitment sources, respectively. GOLD-stage 3 to 4 was associated with significantly better guideline adherence compared to GOLD-stage 2 (OR (95% CI) 18.9 (8.37,42.7)). The unadjusted difference between the two recruitment sources was completely explained by degree of airflow obstruction.

**Conclusion:**

Overall guideline adherence was very low for both recruitment sources. We call for increased attention from authorities and healthcare personnel to improve the quality of care given to this patient group.

**Electronic supplementary material:**

The online version of this article (10.1186/s12890-018-0756-8) contains supplementary material, which is available to authorized users.

## Background

Chronic obstructive pulmonary disease (COPD) is the third-leading cause of death worldwide [1]. COPD is characterized by chronic symptoms such as shortness of breath and coughing, and by exacerbations that are associated with a faster decline in lung function, increased socioeconomic burden, and mortality [2–7]. There are several known environmental risk factors, like smoking, occupational exposure, and air pollution, which make effective disease prevention possible [8–11]. Correct diagnosis, treatment, and prevention of its exacerbations have the potential of further reducing mortality, morbidity, and disease burden on the society.

There are several treatment options for COPD, and keeping up to date is time-consuming for health care providers. Reliable and regularly updated guidelines are therefore very valuable for clinicians, and help them provide the best care for their patients at any time. Despite available guidelines, these are not consistently implemented [12–20], and hence, their potential for reducing various aspects of disease burden is not taken advantage of [21, 22].

The Global Initiative for Chronic Obstructive Lung Disease (GOLD) was launched in 1997 in collaboration with the National institute of Health in USA and the World Health Organisation. GOLD is an international organisation of COPD experts that has developed consensus documents for prevention and management of COPD based on the latest research and evidence. In many countries the GOLD recommendations are perceived as guidelines [23].

Evidence from several studies indicate that COPD is substantially under-diagnosed and that there is limited guideline adherence in several countries [12, 24–31]. So far, no systematic study on guideline adherence for COPD has been undertaken in Norway, and none of the mentioned studies from other countries have compared different sampling sources or patient categories. The reason for including both sampling sources was that previous studies have used differing samples and are therefore difficult to compare. Some show results from general practice [24, 27, 30, 31], others from hospital-admitted or outpatient participants [12, 25, 28, 29], and to our knowledge only one study has reported results from a general population [26]. We wanted to evaluate both sampling sources with the same definitions and multivariate analyses to see if the results differed significantly.

The aim of the current study was to estimate and compare the guideline adherence to COPD treatment in hospital-recruited and population-based COPD patients. In addition, we analysed potential predictors of adherence that can help clinicians improve the care they provide.

## Methods

The EconCOPD study was conducted between March 2005 and August 2006 at Haukeland University Hospital, Bergen, Norway. Details on study design, sampling procedures and data collection have been published previously [32]. The Regional Committee for Medical and Health Research Ethics in Western Norway approved the study (REK Vest case number 252.04), and all participants provided written consent. For the current analyses, we have chosen cross-sectional data from the baseline visit of this study, where a post-bronchodilator spirometry was performed and an interview gathered information about gender, age, smoking habits, education, employment status, comorbidities and medication use.

### Study population and design

The participants were either recruited from a follow-up examination in 2003–2004 of the Hordaland County Respiratory Health Survey, a random and representative sample of the population in Hordaland County in 1985 [33], or from hospital registers from the Department of Thoracic Medicine, Haukeland University Hospital. All participants were current or former smokers of ≥2.5 pack years, and were at least 40 years old. Subjects from the population cohort defined as having COPD were designated as “COPD cases”, whereas subjects from the hospital registers were designated as “COPD patients”.

Post-bronchodilator spirometry was performed according to ATS-standards [34]. COPD was defined as having a ratio of the forced expiratory volume in one second (FEV_1_) to the forced vital capacity (FVC) < 0.70 and FEV_1_ < 80% of predicted according to age, sex and height [35]. The GOLD-stages were defined according to post-bronchodilator percent of predicted FEV_1_, stage II being an FEV_1_ of 50–80%, stage III 30–50%, and stage IV < 30%. Control subjects had both FEV_1_ > 80% of predicted and FEV_1_/FVC > 0.7. As the aim of the current analysis was to evaluate COPD treatment, the control subjects were not included in these specific analyses.

Body mass index (BMI) was defined as weight per squared height (kg/m^2^). Comorbid conditions were defined by confirmative answers to questions from the Charlson Comorbidity Index [36]. Dyspnoea was defined as grade 1 when experiencing dyspnoea walking up 2 flights of stairs, grade 2 when experiencing dyspnoea walking at level ground, and grade 3 when experiencing dyspnoea while at rest. One pack year of tobacco smoking was smoking 20 cigarettes daily for one year.

### Guideline adherence

The main outcome was guideline adherence in population- and hospital-recruited COPD patients. At the time of data collection, the most recent GOLD document had been published in 2001, with an update in 2004 [37]. Based on the 2004 GOLD document we defined treatment as guideline-adherent when the patient had received smoking cessation advice from a doctor, influenza vaccination the preceding year, and correct pharmacological treatment. The latter was stratified by spirometric grade of obstruction. Correct pharmacological treatment was defined as short-acting muscarinic antagonists or beta agonists (SAMA/SABA) and long acting muscarinic antagonists or beta agonists (LAMA/LABA) to all (i.e. no under-utilization), no inhaled corticosteroids (ICS) to GOLD-II participants and no double medication within same drug group (i.e. no over-utilization). ICS was allowed, but not mandatory in GOLD stage III and IV. Since the recommended pharmacological treatment in stage III and IV was identical, we have analysed these patients together. Considering the robust positive effect of rehabilitation [38, 39], we also analysed rates of receiving rehabilitation in the different sampling sources. Rehabilitation was defined as participation in a rehabilitation programme, or visits to an authorized physiotherapist the last 3 months.

### Statistical analyses

A priori power calculations for the EconCOPD-study were performed based on the main objectives of the study. For the analyses regarding healthcare utilization, a sample size of 95 participants in each COPD-group was needed, whilst for the analyses of costs a sample size of 85 participants in each group was needed (aiming for a β-value of 20% and significance level of 5%).

Bivariate comparison of correlates by participant source and outcomes were performed using Kruskal-Wallis, Chi-Squared, Students T, and Wilcoxon rank-sum tests, as appropriate, after evaluating the distribution of the data. For the bivariate comparison of guideline adherence stratified on recruitment source *and* GOLD-stage, we also performed a trend test ranking GOLD-stage 2 in the population-based sample as number 1, GOLD-stage 2 in the hospital-recruited sample as number 2, GOLD-stage 3–4 in the population-based sample as number 3, and finally, GOLD-stage 3–4 in the hospital-recruited sample as number 4. Multivariate logistic regression analysis was performed with the dichotomous outcome overall guideline-adherence, adjusting for sampling source, gender, age, education, smoking habits, dyspnoea, doctor’s diagnosis of asthma, comorbidities, and FEV_1_ in percent predicted. Additional multivariate logistic regression analyses were performed to investigate possible predictors for receiving influenza vaccination and rehabilitation. For all analyses, we used Stata SE version 15.1 (StataCorp, Tx, USA).

## Results

### Characteristics

In total, 90 COPD-cases and 245 COPD-patients provided complete information at the baseline interview. The response rate was 81 and 79%, respectively, for these two sampling sources. The characteristics and number of prescribed medicines for the population- and hospital- recruited COPD-cases are summarized in Table 1. The hospital recruited COPD-patients were significantly older, had lower BMI, more severe airflow obstruction, more respiratory symptoms and comorbidities, and used more prescribed drugs than the population-based COPD-cases. There was no difference in gender, educational level, or pack years smoked between COPD patients from either of the sampling sources, but there were significantly more current smokers in the population sample than in the hospital sample.

**Table 1:** Characteristics of hospital- and population-recruited COPD cases in the EconCOPD-study

	Population based COPD cases	Hospital recruited COPD patients
N	90	245
Female sex, %	34.4	40.0
Age, mean (SD) years	64.1 (10.4)	67.8 (9.5) **
BMI, mean (SD) kg/m2	26.2 (4.5)	25.1 (5.0) *
Education		
Primary, %	38.9	36.7
High School, %	38.9	48.6
University, %	22.2	14.7
Smoking habits		
Current smokers, %	45.6	32.2 *
Pack year, mean (SD)	31.1 (34.1)	31.8 (29.8)
FEV1, mean (SD) %	64.7 (14.4)	47.1 (17.0) **
GOLD-stage**		
GOLD II, %	84.4	48.2 **
GOLD III, %	10.0	33.9 **
GOLD IV, %	5.6	18.0 **
Respiratory symptoms, %	74.4	95.9 **
Dyspnoea at level ground %	6.7	38.1 **
Cough, %	47.8	64.5 **
Phlegm, %	48.9	62.9 *
Number of comorbidities, mean (SD), p50 (IQR)	1.2 (1.5), 1 (2)	1.9 (1.8), 1 (3) **
Number of prescribed drugs, mean (SD) p50 (IQR)	3.0 (2.9), 2.5 (5)	6.3 (4.0), 6 (6) **

* = *p*-value < 0,05. ** = p-value < 0,01 by Kruskal-Wallis and Fishers´ exact test. SD = standard deviation. p50 = median. *IQR* interquartile range

Low-threshold dyspnoea = Dyspnoea when walking at normal pace on flat ground, but not at rest

Number of prescribed drugs includes all medication irrespective of cause

### Treatment by recruitment source and GOLD-stages

We found significant differences between COPD cases and COPD patients for all aspects of guideline adherence (Table 2). The overall adherence in the population-based sample was as low as 6.7% vs 29.8% for the hospital-recruited sample. Awareness of having COPD, in spite of being informed of having COPD when performing spirometry, varied from 34.4% in the population-based sample to 85.5% in the hospital-recruited sample. Adherence to influenza vaccination the preceding 12 months, rehabilitation, smoking cessation advice, and correct pharmacological treatment were all significantly different between the two sampling sources, and consistently higher in the hospital-recruited sample.

**Table 2:** COPD-treatment in percentage, stratified by recruitment source, results from unadjusted statistical comparison in the EconCOPD-study

	Population based COPD cases *N* = 90	Hospital recruited COPD patients *N* = 245
COPD awareness (incl. Chronic bronchitis and emphysema)	34.4	86.5 *
Influenza vaccine preceding 12 months	34.4	70.6 *
Rehabilitation	5.6	16.7 *
Smoking cessation advice (if still smoking)	82.5	96.2 *
Guideline adherent pharmacological treatment	10.0	35.5 *
Overall guideline adherence	6.7	29.8 *

*= *p*-value < 0.05 by Fishers´ exact test

Rehabilitation = joined a rehabilitation-program or been in contact with a physiotherapist during the last 3 months

Correct pharmacological treatment = reliever therapy and maintenance therapy and not “overdosed”. In addition, no kind of ICS to GOLD2-participants

“Overdosed” = taking same kind of inhaled medicine through 2 different devices

Guideline adherent pharmacological treatment: SAMA/SABA and LAMA/LABA (all). No ICS in GOLD II, no double medication within same drug group

Overall guideline adherence = receiving correct pharmacological treatment and influenza vaccine and smoking cessation advice if still smoking

Comparing the treatment by GOLD-stage *and* source of recruitment, the unadjusted analyses only showed significant differences between the cases and patients within GOLD stage 2 (Table 3). The different components of medical treatment for the two samples of GOLD 2-participants differed significantly, with the only two exceptions being ICS and oral b2-agonist. Adherence to advice on influenza vaccination was significantly different between the population-based and the hospital-recruited samples in GOLD stage 2. Only 2 individuals with GOLD stage 2 disease were guideline adherent and had received pulmonary rehabilitation the preceding 3 months, whereas 17 individuals with stage 3/4 disease had received this treatment. We then ranked participants by GOLD-stages and recruitment source in the order: GOLD stage 2 cases, GOLD stage 2 patients, GOLD stage 3/4 cases, GOLD stage 3/4 patients. Trend tests on this ranking revealed that for short-acting and long-acting bronchodilators, combination therapy, overall correct pharmacological treatment, influenza vaccination, rehabilitation, anti-smoking advice, and overall guideline adherence the trend was significant for increasing adherence with increasing rank (results not shown, all *p* < 0.05).

**Table 3:** COPD-treatment in percentage, stratified by recruitment *source and* GOLD-stage, results from unadjusted statistical comparison in the EconCOPD-study

	GOLD 2		GOLD 3–4	
	Population-based COPD cases (*n* = 76) Rank 1	Hospital-recruited COPD patients (*n* = 118) Rank 2	Population-based COPD cases (*n* = 14) Rank 3	Hospital-recruited COPD patients (*n* = 127) Rank 4
SAMA	9.2	39.0 **	42.9	56.7
SABA	23.7	45.8 **	42.9	55.9
LAMA	1.3	15.3 **	14.3	28.4
LABA	6.6	17.8 *	14.3	13.4
ICS	6.6	12.7	7.1	8.7
LABA+ICS in one device	18.4	50.0 **	57.1	71.7
Methylxantines	0.0	13.6 **	0.0	17.3
Oral beta2-agonist	4.0	5.1	0.0	7.9
Prednisolone or methylprednisolone	4.0	17.0 **	7.1	18.1
-only short period	2.6	5.1	7.1	8.7
Guideline adherent pharmacological treatment	4.0	7.6	42.9	61.4
Self-reported doctors´ diagnosis of COPD, emphysema or chronic bronchitis	27.6	80.5**	71.4	92.1*
Influenza vaccine preceding 12 months	30.3	60.2**	57.1	80.3
Rehabilitation	5.3	11.0	7.1	22.1
Anti-smoking advice (if still smoking) ***	82.9	93.6	80.0	100
Overall guideline adherence	1.3	5.9	35.7	52.0
Overall guideline adherence AND rehabilitation, *N *(%)	1 (1.3)	1 (0.9)	0 (0)	17 (13.4)

* = p-value < 0.05, and ** = p-value < 0.01, by Fishers´ exact test

***lower N (low number of participants)

SAMA = inhaled shortacting muscarinergic agonist. SABA = inhaled shortacting beta2 agonist. LAMA = inhaled longacting muscarinergic agonist. LABA = inhaled longacting beta2 agonist. ICS = only ICS (inhaled corticosteroids) in the device

### Guideline adherence and predictors

Neither gender, level of education, respiratory symptoms (with the exception of the dichotomous variable of having dyspnoea or not in the population sample), self-reported asthma, age, BMI, income, pack years, or number of comorbid conditions was associated with guideline-adherence (Tables 4 and 5). Decreasing FEV_1_ (GOLD stage, FEV_1_% predicted) and increasing number of prescribed drugs were statistically associated with increased adherence.

**Table 4:** Percentage of overall guideline adherence in population based COPD and hospital recruited COPD patients by the categorical variables gender, education, respiratory symptoms and asthma diagnosis

		Population based COPD cases *N* = 90	Hospital recruited COPD patients *N* = 245
Sex	Female	9.7	29.6
	Male	5.1	29.9
Education	Primary	8.6	28.9
	High school	5.7	27.7
	University	5.0	38.9
Lung function	GOLD II	1.3*	5.9*
	GOLD III and IV	35.7*	52.0*
Respiratory symptoms	Dyspnoea	14.0*	32.5
	Cough	9.3	29.1
	Phlegm	4.6	28.6
Doctor’s diagnosis of asthma	No	6.5	26.7
	Yes	7.1	32.8

*= p-value < 0.05 by Fisher’s test on intra-group comparison

Guideline adherence = reliever therapy and maintenance therapy and not “overdosed”. In addition, no kind of ICS to GOLD2-participants

**Table 5:** Mean differences of age, BMI, income, tobacco exposure, FEV_1_, number of comorbid conditions, and number of drugs in use between guideline adherent and nonadherent COPD subjects, stratified by recruitment source

	Population based COPD cases, mean difference adherent - nonadherent	Hospital recruited COPD patients, mean difference adherent - nonadherent
Age (years at baseline)	11.1	−0.1
BMI (kg/m^2^)	−2.3	−0.4
Income (NOK yearly income)	401,898	− 9539
Pack years ((cig/day*years smoked)/20)	−3.9	−0.2
FEV_1_ (post-bronchodilator % of predicted)	−29.9**	− 16.5**
Number of comorbid conditions (mean)	0.8	0.1
Number of drugs in use (mean)	3.6**	1.0

**= *p*-value < 0.01 by Mann-Whitney test on intra-group comparison

In bivariate logistic regression analyses on pooled data, several covariates were significantly associated with better guideline adherence (Table 6). Belonging to the hospital sample had an OR (95% CI) of 5.9 (2.5–14.2) for correct guideline adherence compared to the population sample. Compared to GOLD-stage 2, GOLD-stage 3 *and* 4 (analysed together) was associated with a substantially higher possibility of receiving correct treatment, OR 23.6 (10.8–51.5), highly significant with *p* < 0.001. Being an ex-smoker and having dyspnoea were also both associated with significantly better guideline adherence, OR 2.2 (1.3–4.0) and 4.3 (2.0–9.3), respectively.

**Table 6:** Bivariate and multivariate logistic regression for guideline adherence (odds ratios (OR), and 95% CIs in brackets) to COPD treatment in hospital- and population-recruited patients in the EconCOPD-study

	Bivariate	Multivariate
Population-based COPD cases	ref	ref
Hospital-recruited COPD patients	5.9** [2.5,14.2]	2.4* [0.9,6.5]
Gender		
Male	ref	ref
Female	1.1 [0.7,1.9]	1.4 [0.7,2.7]
Age, 10 yrs. increment	1.2 [0.9,1.6]	1.1 [0.8,1.6]
GOLD-stage		
Stage 2	ref	ref
Stage 3 and 4	23.6** [10.8,51.5]	19.0** [8.4,43.0]
Smoking status		
Current smoker	ref	ref
Ex-smoker	2.2** [1.3,4.0]	1.4 [0.7,2.9]
Education		
Primary School	ref	ref
High School	1.0 [0.6,1.7]	1.3 [0.6,2.7]
University	1.2 [0.6,2.5]	2.0 [0.8,5.1]
Dyspnoea		
No dyspnoea	ref	ref
Dyspnoea, any grade	4.3** [2.0,9.4]	1.6 [0.6,4.3]
Doctors diagnosis asthma		
No	ref	ref
Yes	1.6* [1.0,2.6]	0.8 [0.4,1.6]
Number of comorbid conditions (mean)	1.1 [1.0,1.3]	1.0 [0.9,1.3]
*N*	335	335

*: *p* < 0.10

**: *p* < 0.05

With one exception, all the predictors associated with better guideline adherence in bivariate analyses lost their significance in multivariate regression analyses (Table 6). The exception was GOLD-stage 3 and 4 which gave OR 19.0 (8.4–43.0) with a *p*-value < 0.001. Belonging to the hospital sample yielded an OR of 2.4 (0.9–6.5), with *p* = 0.095.

Additional file 1: E-Table 1 and Additional file 2: e-Table 2 shows the results of multivariate logistic regression analyses for possible predictors of receiving influenza vaccination and pulmonary rehabilitation. For influenza vaccination, we saw that belonging to the hospital sample, being of higher age, and having more severe airflow obstruction were all associated with significantly higher OR, i.e. these variables predicted better vaccination coverage. For participation in rehabilitation, being an ex-smoker was the only significant predictor. Having an airflow obstruction categorized as GOLD-stage 3 and 4 gave a borderline significance with OR (95% CI) 1.9 (0.9–3.9), and *p*-value 0.08, implying higher inclusion in rehabilitation programmes for these patients.

## Discussion

We have found that, on average, the overall guideline adherence to COPD treatment for population-based COPD cases was 6.7%, which was significantly lower compared to the 29.8% overall guideline-adherence amongst hospital-recruited COPD-patients. Adherence to pharmacological treatment guidelines was 10.0 and 35.5%, for the population-based and hospital-recruited participants, respectively. In multivariate regression analyses we found that having GOLD-stage 3 and 4 was associated with significantly better guideline adherence, and a trend of more adherence in hospital treated patients. The grand majority of all participants who still smoked had been given smoking cessation advice. The observed higher rate of current smoking amongst the COPD cases is most likely due to significantly less symptoms in this group compared to the hospital-recruited COPD patients, leading to fewer quitters. Additionally, there were significantly fewer COPD cases that had received smoking cessation advice.

The lack of adherence was significantly lower in the general population, and multivariate regression analyses indicated that the main cause for this was more severe COPD in the hospital-recruited sample. This finding highlights the importance of improving the quality of COPD treatment of milder cases of COPD, that are treated by general practitioners. In Denmark, Lange et al. showed that after implementation of an educational programme on COPD for general practitioners, the quality of care increased significantly with more use of spirometry and more appropriate use of ICS [24]. In Swedish general practice, the management of COPD patients has been proven inadequate [40]. The current study is by design unable to identify the “perpetrator” of poor guideline adherence, as both patients themselves, healthcare practitioners, and effectiveness of available treatment will likely influence adherence. But both health care personnel and the authorities should give this patient group greater attention through increasing the level of knowledge of the disease, and through simplification of access to and implementation of treatment guidelines. When performing spirometry, all the participants who appeared to have COPD were given notice about their status. Even so, only 34.4% of the cases answered that they were aware of having COPD when asked during the baseline interview. There might be several explanations for this, one being the ability of the study doctor or nurse to inform in a manner that made the participants understand their results. And another explanation might be that COPD is a stigmatising disease which the participants wished not to recognize.

The low COPD guideline adherence in our study has also been observed in other countries [12, 16, 18, 20, 28–31]. This deficiency does not only imply a precarious treatment of inferior quality for the patients, it can also have juridical consequences for the health personnel responsible for the treatment. By Norwegian law, the reason of failure to adhere to guidelines must be recorded in the patient journal. The current study is the first to demonstrate overall guideline adherence in COPD in Norway. Previous studies have looked at the use of ICS and also other maintenance drugs and medication during acute exacerbations [41–43]. These studies showed over-prescription of ICS to COPD-patients, and that objective diagnostic tests to evaluate if the patients really are having an exacerbation are rarely recommended. The proportion of participants who both received overall guideline adherent treatment *and* pulmonary rehabilitation was 13% among the subjects likely to have more severe disease. This is particularly alarming, given the evidence for positive effect of rehabilitation [38, 39].

In 2014, the Norwegian Heart and Lung patient organisation performed a survey that showed that amongst the institutions responsible for the education of medical doctors or psychologists, 71% used COPD guidelines, but only 24% of the institutions educating nurses and physiotherapists [44]. A structured education of health care personnel on how to implement guidelines is warranted, along with a possible establishment of incentives for the health care personnel who do adhere to the guidelines.

The main strength of this study is the ability to provide overall guideline adherence in both population-based and hospital recruited COPD patients. The observed difference between these sampling sources was completely explained by disease severity. Diagnoses were confirmed by state-of-the-art spirometry, and we have chosen to emphasize clinically significant disease by excluding subjects with FEV1 above 80% of predicted. Additionally, our study is the first of its kind to be conducted in Norway. Nevertheless, there are some possible limitations that deserve mentioning. The sample size for the population-recruited COPD cases is of borderline magnitude, though this only serves to give more conservative results than what might be true for a larger population-based sample. Further on, the participants were recruited from the city of Bergen in Western Norway and 11 surrounding municipalities, and this might have an influence on the external validity of the study. However, a comparison between national Norwegian survey data and the original cohort from which the EconCOPD-study recruited from, showed no discrepancies [45]. Additionally, we lack information of the role of oxygen as we did not perform arterial blood gas samples. Further, some of those invited to participate did decline the invitation. Analyses were made to evaluate if the non-responders would have changed our results, but there were no significant differences in gender or FEV_1_ percentage of predicted between non-responders and responders [45, 46]. Finally, some years have passed by since the data in this study was collected, but the epidemiology of COPD and its disease mechanisms are the same, no major drug groups have been developed since undertaking the EconCOPD-study, and the most effective health care interventions are still smoking cessation, rehabilitation and oxygen supplement on the clinically appropriate indication.

The guideline adherence for treatment of COPD was disappointingly low. This finding should be taken as an encouragement to healthcare practitioners and decision makers, because it highlights that the potential for improvement for these patients is considerable. We need accessible guidelines for healthcare workers, drug devises that facilitates compliance, accessible rehabilitation facilities, and vaccination programmes to ensure that COPD patients are offered the best care available.

## Conclusions

We conclude that COPD patients receive guideline adherent treatment in 6.7 and 29.8% of the cases from a general population and from a hospital register, respectively, and that the difference between the two samples is explained by grade of airflow obstruction. We call for increased attention from authorities and health care personnel to improve the quality of care given to this patient group.

## Additional files

Additional file 1: Multivariate logistic regression for receiving influenza vaccination (odds ratios (OR), and 95% CIs in brackets) in hospital- and population-recruited patients in the EconCOPD-study. Table containing ORs (odds ratios) from multivariate regression analysing whether there were any predictors for receiving influenza vaccination. (RTF 64 kb)

Additional file 2: Multivariate logistic regression for participating in pulmonary rehabilitation (odds ratios (OR), and 95% CIs in brackets) in hospital- and population-recruited patients in the EconCOPD-study. Table containing ORs from multivariate regression analysing whether there were any predictors for participating in pulmonary rehabilitation. (RTF 61 kb)

## Abbreviations

ATS: American Thoracic Society
BMI: Body mass index
CI: Confidence interval
COPD: Chronic obstructive pulmonary disease
EconCOPD: Economics of COPD-study
FEV_1_: Forced expiratory volume in 1 s
FVC: Forced vital capacity
GOLD: Global initiative for chronic obstructive lung disease
ICS: Inhaled corticosteroid
LABA: Long-acting beta agonist
LAMA: Long-acting muscarinic antagonist
N: Number of participants
NOK: Norwegian krone
OR: Odds ratio
p50: Median
REK: The Regional Committee for Medical and Health Research Ethics
SABA: Short-acting beta agonist
SAMA: Short-acting muscarinic antagonist
SD: Standard deviation
