# Supplementary material for: Guideline adherence in hospital recruited and population based COPD patients
Source: BMC Pulm Med. 2018 Dec 20;18:195. doi: 10.1186/s12890-018-0756-8 (PMC6302492; doi:10.1186/s12890-018-0756-8)
Supplement: Supplementary file 1 — Multivariate logistic regression for receiving influenza vaccination (odds ratios (OR), and 95% CIs in brackets) in hospital- and population-recruited patients in the EconCOPD-study. Table containing ORs (odds ratios) from multivariate regression analysing whether there were any predictors for receiving influenza vaccination. (RTF 64 kb) [file 12890_2018_756_MOESM1_ESM.rtf]

E-Table 1: Multivariate logistic regression for receiving influenza vaccination (odds ratios (OR), and 95% CIs in brackets) in hospital- and population-recruited patients in the EconCOPD-study.

	Multivariate OR [95% CI]	
Population-based COPD cases	ref	
Hospital-recruited COPD patients	2.9
[1.6,5.4]	
Male	ref	
Female	1.4
[0.8,2.6]	
Age, 10 yrs increment	2.0
[1.5,2.6]	
GOLD-stage 2	ref	
GOLD-stage 3 and 4	2.9
[1.7,5.2]	
Current smoker	ref	
Ex-smoker	1.6
[0.9,2.7]	
Primary School	ref	
High School	1.4
[0.8,2.6]	
University	1.9
[0.9,4.2]	
No dyspnea	ref	
Dyspnea	1.2
[0.6,2.3]	
No asthma	ref	
Doctors diagnosis asthma	0.7
[0.4,1.2]	
Number of comorbid conditions (mean)	1.1
[1.0,1.3]	
N	335	
Exponentiated coefficients; 95% confidence intervals in brackets
