# Supplementary material for: Guideline adherence in hospital recruited and population based COPD patients
Source: BMC Pulm Med. 2018 Dec 20;18:195. doi: 10.1186/s12890-018-0756-8 (PMC6302492; doi:10.1186/s12890-018-0756-8)
Supplement: Supplementary file 2 — Multivariate logistic regression for participating in pulmonary rehabilitation (odds ratios (OR), and 95% CIs in brackets) in hospital- and population-recruited patients in the EconCOPD-study. Table containing ORs from multivariate regression analysing whether there were any predictors for participating in pulmonary rehabilitation. (RTF 61 kb) [file 12890_2018_756_MOESM2_ESM.rtf]

E-Table 2: Multivariate logistic regression for participating in pulmonary rehabilitation (odds ratios (OR), and 95% CIs in brackets) in hospital- and population-recruited patients in the EconCOPD-study.

	Multivariate OR [95% CI]	
Population-based COPD cases	ref	
Hospital-recruited COPD patients	2.3
[0.8,6.4]	
Male	ref	
Female	1.4
[0.7,2.9]	
Age, 10 yrs increment	0.98
[0.7,1.4]	
GOLD-stage 2	ref	
GOLD-stage 3 and 4	1.9
[0.9,3.9]	
Current smoker	ref	
Ex-smoker	5.0
[1.8,13.6]	
Primary School	ref	
High School	1.3
[0.6,2.7]	
University	0.5
[0.1,1.6]	
No dyspnea	ref	
Dyspnea	1.2
[0.4,3.4]	
No asthma	ref	
Doctors diagnosis asthma	0.8
[0.4,1.6]	
Number of comorbid conditions (mean)	1.0
[0.8,1.2]	
N	335	
Exponentiated coefficients; 95% confidence intervals in brackets
